# Chios Mastic Gum: Chemical Profile and Pharmacological Properties in Inflammatory Bowel Disease: From the Past to the Future

**DOI:** 10.3390/ijms241512038

**Published:** 2023-07-27

**Authors:** Roberta Ottria, Ornella Xynomilakis, Silvana Casati, Ezio Abbiati, Giovanni Maconi, Pierangela Ciuffreda

**Affiliations:** 1 Dipartimento di Scienze Biomediche e Cliniche, Università degli Studi di Milano, 20157 Milan, Italy; ornella.xynomilakis@unimi.it (O.X.); silvana.casati@unimi.it (S.C.); giovanni.maconi@unimi.it (G.M.); pierangela.ciuffreda@unimi.it (P.C.); 2 Phytoitalia S.r.l., Via Gran Sasso, 37, Corbetta, 20011 Milan, Italy; ezio.abbiati@phytoitalia.it; 3 Gastroenterology Unit, Luigi Sacco University Hospital, 20157 Milan, Italy

**Keywords:** *Pistacia lentiscus* var. *Chia*, Chios mastic gum, chemical profile, inflammatory bowel disease, IBD, phytochemistry

## Abstract

Chios mastic gum, the product of the tree *Pistacia lentiscus* var. *Chia*, has been used for more than 2500 years in traditional Greek medicine for treating several diseases, thanks to the anti-inflammatory and antioxidant properties of its components. Despite the long-time use of mastic in gastroenterology and in particular in chronic-inflammation-associated diseases, to date, the literature lacks reviews regarding this topic. The aim of the present work is to summarize available data on the effects of *P. lentiscus* on inflammatory bowel disease. A comprehensive review of this topic could drive researchers to conduct future studies aimed at deeply investigating *P. lentiscus* effects and hypothesizing a mechanism of action. The present review, indeed, schematizes the possible bioactive components of mastic gum. Particular care is given to *P. lentiscus* var. *Chia* medicaments’ and supplements’ chemical compositions and their pharmacological action in inflammatory bowel disease.

## 1. Introduction

Over the last few years, plant-based products, and among them, species of the genus *Pistacia,* garnered a lot of attention. Different parts of *Pistacia* species including resin, leaves, fruits, and aerial parts have been traditionally used for a wide range of purposes. The species *P. lentiscus* is most commonly used in different regions, and its resin has been utilized for as long as 5000 years. In Egypt, *P. lentiscus* found application as a preservative, in breath sweetener, or as incense [1]. The chemical investigation of archaeological finds, dated from the Middle Dynastic period to the Late Roman period (1900 B.C.–395 A.D.), evidenced *P. lentiscus* use in the ancient Egyptian embalming process [2]. In Iranian culture, different species, such as *Pistacia vera, Pistacia khinjuk Pistacia terebinthus,* and *Pistacia lentiscus*, found applications for the medical treatment of different conditions and diseases. As indicated in Avicenna’s Canon of Medicine, an encyclopedia of medicine, which encloses the medical knowledge of the Islamic world in XI century, written by Persian physician-philosopher Avicenna, *P. vera* found application in dermatitis and the elimination of fever and headaches [3]. In Makhzan al-Adviyeh, another Persian medical book written in the XII century, is reported how the fruits of *Pistacia atlantica, Pistacia khinjuk,* and *Pistacia terebinthus* had aphrodisiac activity and were used for the treatment of liver, kidney, heart, and respiratory system disorders [4]. Plants belonging to *P. lentiscus* are so far the most commonly used. Resin (mastic) has been used for gastrointestinal diseases in the Mediterranean and Middle East countries for the last 3000 years and continues to have alimentary applications until now. One of the most famous examples is the consumption of pistachio (*P. vera*) [5]. *Pistacia* species, indeed, are not only used for their pharmacological effects but also in the food industry. The resin obtained from *P. vera* is used for gum and as a blood-clotting agent in Europe and the Middle East [6]. Moreover, the *P. terebinthus* fruit has applications as a snack food or in making coffee-like drinks [7].

Due to the long-time application of *Pistacia* derivatives and extracts from ancient times to the present, several preclinical and clinical studies have been performed, and reviews of its application in different diseases have been published. Surprisingly, despite the 3000 years of mastic use in the gastrointestinal field and, in particular, in chronic-inflammation-associated diseases, to date, the literature lacks reviews regarding this topic. Among the different species, *P. vera* and *P. lentiscus* are the main ones studied in the Mediterranean area, highlighting major beneficial and pharmacological effects associated with *P. lentiscus* and, in particular, with the variety *Chia*. Moreover, the literature presents a large variety of *P. lentiscus* preparations, assessed for their pharmacological effects, derived from different parts of the plant and consequently characterized by the presence of different families of potentially active compounds. Finally, some articles have been published in recent years, with a focus on the ethnopharmacology, phytochemistry profile, and pharmacological properties of *P. lentiscus* var. *Chia*.

Data available from the literature are too confounding to enable researchers to fully comprehend the mechanism of action by which Pistacia exerts its beneficial effects, also considering the numerous medications or bioactive food supplements based on Pistacia extracts, as well as the various species and varieties of Pistacia. Moreover, the complex chemical composition of *P. lentiscus* preparations makes it difficult for researchers to hypothesize a possible mechanism of action and concentrate their efforts on specific biochemical pathways. In addition, despite the traditional use of mastic in gastroenterology and in chronic inflammation, to date, the literature lacks a comprehensive review on this topic. To fill this gap, this review aims at schematizing the possible bioactive components of mastic gum. Particular care is given to *P. lentiscus* var. *Chia* medicaments’ and supplements’ chemical compositions. *P. lentiscus’* pharmacological action in inflammatory bowel disease is also explored.

## 2. Taxonomy and Geographical Distribution

**Figure 1:**
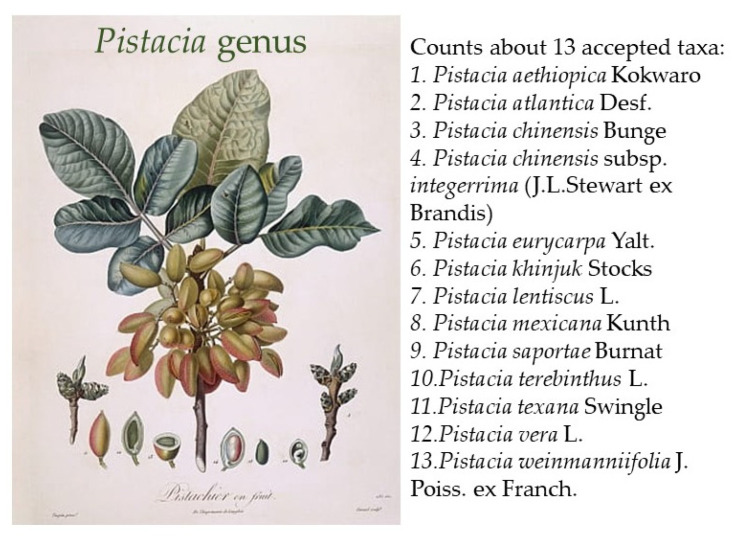
*Pistacia* species; Pistacio Illustration by Pierre Antoine Poiteau from Flore des Antilles circa 1808.

Linnaeus was the first to establish the genus *Pistacia*, recognizing six species as reported in his manuscript “Species Plantarum” (Linné Carl von Stearn WT 1753). The genus *Pistacia* belongs to the *Anacardiaceae*, a cosmopolitan family that comprises about 70 genera and over 600 species. The actual data report of the genus *Pistacia* lists 13 globally accepted species according to the WFO plant list (WFO Dic 2022) (Figure 1). With a wide distribution, the native range of this genus is Eurasia, Africa, and America (POWO) [8]. Species members are evergreen or mainly deciduous, and most of them are known to be strictly dioecious, which means that male and female flowers are housed on different plants with several exceptions such as *P. chinensis* and *P. atlantica* [9].

Many *Pistacia* species yield resin to some degree, and two of them, namely *P. atlantica* and *P. lentiscus* var. *Chia,* are the major sources of resin that can be collected and used and that have been commercialized for centuries. In particular, *P. lentiscus* var*. Chia* (Mastic tree), is an evergreen shrub [10] with a strong characteristic aroma and green leaves. Today, the *P. lentiscus* var*. Chia* is cultivated especially on the Greek island of Chios in the northern Aegean Sea for its fragrant and aromatic resin, presumably due to a unique combination of climatic factors and ground conditions. This resin is called Chios mastic gum (CMG), or mastic (known as the “tears of Chios”), an exudate obtained from the stem as a protective reaction against unfavorable environmental conditions [11]. The biosynthesis of the secondary metabolites, indeed, is directly related to the environmental conditions [12] leading to different compositions of the secreted materials also among the different seasons. *P. lentiscus* var*. Chia* is by far the second economically important species in the *Pistacia* genus; the first is *P*. *vera* for pistachio consumption because its fruits, resins, and leaves have a long tradition in folk medicine and therapeutic properties, known since ancient Greece. Mastic is extracted after shallow incisions are made on the trunk and the thicker branches of the plant, repeated three times a year from July to September. The resin coagulation lasts about 15 days, and CMG is collected by hand.

## 3. Phytochemistry

Detailed phytochemical studies on *P. lentiscus* have led to identifying a number of secondary metabolites in the bark of stems and roots, fruits, leaves, essential oils, and mastic resin such as terpenes, flavonoids, tannins, steroids, and fatty acids. These substances have shown interesting bioactivities in vivo or in vitro, and some of them are protected by a patent. *P. lentiscus* products, nowadays, have also a wide range of uses in food supplements and cosmetics [13] due to the beneficial effects related to flavonoids, polyphenols, and phenolic acids.

**Figure 2:**
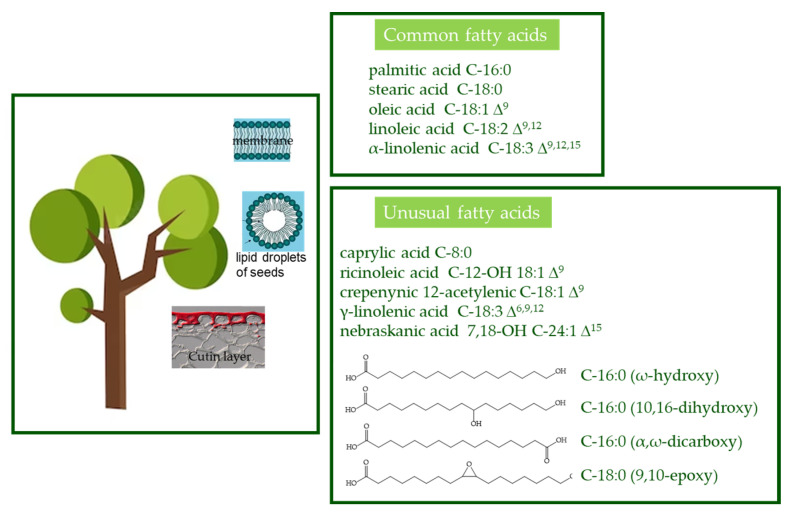
Localization of major types of fatty acids present in plants. Membranes are almost exclusively composed of common fatty acids, while lipid droplets of seeds and cutin are the major sites for unusual fatty acids.

***Fatty acids.*** Fatty acids are one of the most ubiquitous natural products in the form of complex mixtures of liquids or low-melting-point solids hard to separate and purify. In plants, fatty acids are components of glycerolipids (galactolipids, sulfolipids, phospholipids, betaine lipids, and triacylglycerols), sphingolipids, and extracellular lipids (cuticular waxes and lipid polyesters) [14]. They have a myriad of functions such as structural components of the membranes or cuticles [15], carbon and energy storage, and signal transduction [16]. Although more than 450 different fatty acids are known in the plant kingdom [17], only palmitic acid (C-16:0), stearic acid (C-18:0), oleic acid (C-18:1 Δ^9^), linoleic acid (C-18:2 Δ^9,12^), and α-linolenic acid (C-18:3 Δ^9,12,15^) [16], called “common fatty acids”, are widely present in membrane lipids and in triacylglycerols. Conversely, “unusual fatty acids” include those containing fewer than 16 or more than 18 carbon atoms, variable double bond positions and cis/trans orientations, and such other side chain functional groups (Figure 2) and are often found as major components of seed oils. Unusual fatty acids can also be found in a polymerized form in cutin, a lipid polyester found near the epidermal surface of plants. The unusual vegetable fatty acids have long intrigued basic and applied researchers due to their biological significance and properties that impart functionality to vegetable oil.

In *Pistacia* species, the major fatty acid in seed and kernel is oleic acid. Other fatty acids identified in leaves are long- and medium-chain saturated fatty acids such as palmitic, myristic, stearic, and long-chain poly-unsaturated fatty acids (PUFAs) such as linolenic, arachidonic, and pentadecanoic acids characterized by a composition variability linked to the season and site of cultivation, with higher PUFAs levels in the coldest season and places [18,19,20]. In the last few decades, the oil from *P. lentiscus* fruits has been recommended as a new source for the production of vegetable oils based on the high amount of mono-unsaturated and omega-3 fatty acids like oleic acid and linolenic acid and high quantity of phytosterols like *β*-sitosterol [20,21].

**Figure 3:**
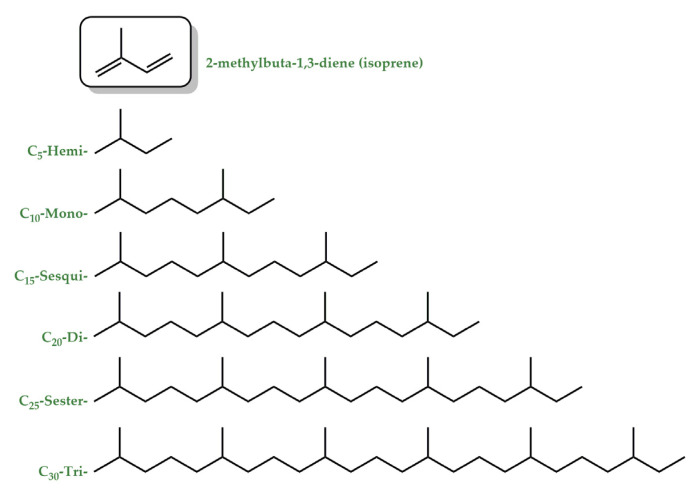
Hydrocarbon terpenes: categories of the sequential combining of basic five-carbon units.

***Terpenes.*** Terpenes constitute a large and diverse class of natural products with >55,000 known compounds [22]. The basic structure of terpenes is an isoprene (2-methylbuta-1,3-diene) unit, and based on the number of isoprene units in a molecule, terpenes can be classified as mono-(C10), sesqui-(C15), di-(C20), sester-(C25), and triterpenes (C30) (Figure 3) [23]. They are abundantly found in fruits, vegetables, and flowers [24], and their concentration is generally high during and immediately following flowering [25]. They are responsible for the typical fragrance of many plants and function as info chemicals, attractants, or repellents. At high concentrations, indeed, they can be toxic and are thus an important weapon against herbivores and pathogens [25,26]. Terpenes are biochemically transformed by adding functional groups containing heteroatoms to form terpenoids. The terms terpene and terpenoid are often used interchangeably [27]. Monoterpenes and most of the sesquiterpenes are volatile compounds and are primary components of essential oils (EOs), while diterpenes are the major components of resins due to their low volatility and high boiling points. EOs, less than 5% of the vegetable dry matter [28], are concentrated liquids of complex mixtures that can be extracted from all the different parts of plants or from the whole plant for a single botanical source [29,30]. Chemically, EOs are a rich mixture of numerous bioactive chemical components such as terpenes and terpenoids as major components and phenylpropanoids and others as minor constituents [31,32].

*P. lentiscus* EOs demonstrated antioxidant properties in different in vitro experiments depending on the season and site of cultivation. The extract composition has been investigated by using GC/MS, and a large number of aroma compounds have been identified, with monoterpene and sesquiterpene oxygenated hydrocarbons as the most abundant ones [33,34]. Moreover, the antibacterial and antifungal activities of *P. lentiscus* EOs have been recently demonstrated with promising results [35]. Finally, *P. lentiscus* EOs incorporated in liposomes accumulate in the derma and are able to counteract damages induced by oxidative processes and promote beneficial effects on lesion regeneration and healing [36].

**Figure 4:**
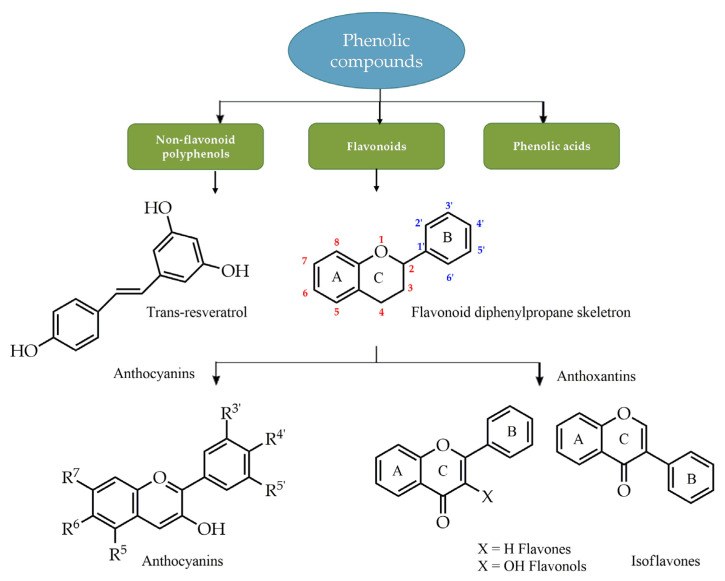
Classification of phenolic compounds.

***Phenolic compounds.*** Polyphenols are natural substances ubiquitously present in fruits and vegetables, as well as beverages obtained from plants such as tea, red wine, and olive oil. They are a group of small organic molecules synthesized by plants as secondary metabolites [37] and are generally involved in defense against ultraviolet radiation or aggression by pathogens [38,39]. More than 8000 polyphenolic compounds have been identified in various plant species with a broad range of biological activities, such as antioxidant, anti-inflammatory, cardioprotective, and neuroprotective, conferred by the numerous phenolic hydroxyl groups on the aromatic ring [40]. Phenolic compounds are subdivided into groups (Figure 4) by the number of phenolic rings and of the structural elements that link these rings [41]. The largest group of polyphenols are flavonoids, widely distributed in the leaves, seeds, bark, and flowers of plants. Their skeletal structure consists of an aromatic ring condensed to a heterocyclic ring, attached to a second aromatic ring. Due to the structural differences, flavonoids are classified into anthocyanins, glycosylated derivatives of anthocyanidin, present in colorful flowers and fruits, and antoxantins, colorless compounds further divided into several categories including flavones, flavans, flavonols, flavanols, and isoflavones (Figure 4). Due to their diverse therapeutic effects, they are a focus of interest in traditional medicine and drug development.

The amount and composition of phenolic compounds in *Pistacia* depend on species, geographical origin, sampling period, plant part, and the type of solvent used during the extraction. *P. lentiscus* leaves, methanolic extracts, and vegetative period lead to the highest and richest phenolic extracts [42,43]. *P. lentiscus* extracts’ biological activity as antioxidant, antimicrobial, and anti-inflammatory agents has also been explored due to their use in traditional medicines. Barbouchi and co-workers [44] demonstrated the link between its free radical formation prevention and the high content of phenolic compounds, in particular gallic acid and their galloyl derivatives, by a significant positive correlation between the antioxidant test results and the number of total phenols. The presence of flavonoid compounds in methanol extracts demonstrated interesting antimicrobial potential against Gram-positive and Gram-negative bacteria [45]. Anti-inflammatory activity, instead, is probably due to the presence of quercetin-3-glucoside, the major flavonol in *P. lentiscus*, besides the presence of antioxidants such as flavonoids and polyphenols [46].

### CMG Composition

CMG, obtained exclusively from *P. lentiscus* var. *Chia*, has been used for more than 2500 years thanks to the anti-inflammatory and antioxidant properties of its components [47]. To date, few studies have been conducted on the elucidation of the chemical composition of CMG, and even fewer on the factors influencing it. A possible reason for this is the difficult handling of the resin, probably due to the poor solubility of the polymer but also to the high concentration of triterpenes present in the gum. CMG is highly insoluble in water, and the most appropriate and commonly used solvents for dissolving the resin are non-polar solvents such as diethyl ether, dichloromethane, and ethyl acetate.

**Figure 5:**
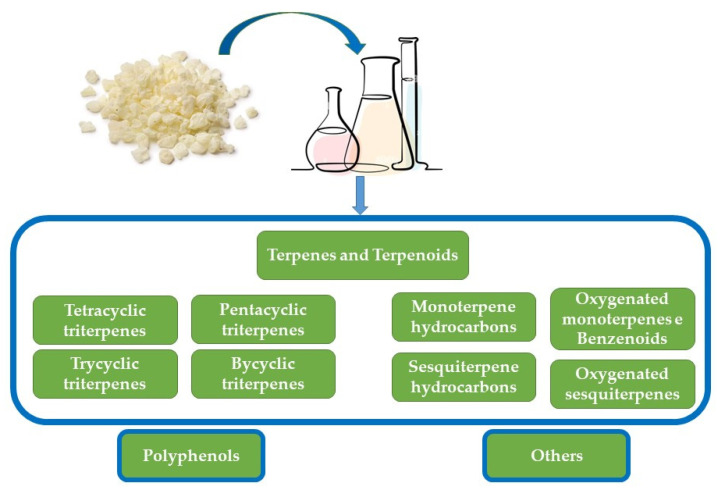
Majors and minor components of CMG.

From a chemical point of view, CMG is a very complex natural resin in which, to date, about 120 chemical compounds have been reported, mainly divided into three categories of substances: the polymer, the volatile fraction (essential oil), and the triterpene content (Figure 5).

Triterpenes, classified into neutral and acidic, constitute the main chemical group of CMG, about 65–70% of the total weight of the resins, and consist mainly of tetracyclic and pentacyclic triterpenes. The main constituents of the acidic fraction are oleanonic acid, moronic acid, 24*Z*-masticadienonic acid, 24*Z*-isomasticadienonic acid, 24*Z*-masticadienolic acid, and 24*Z*-isomasticadienolic acid 18-α-H-oleanonic acid [48,49,50,51,52]. Among the neutral triterpenes, the major compounds were found to be tirucallol, dammaradienone, 28-norolean-12-en-3-one, oleanonic aldehyde, and oleanolic aldehyde.

The volatile compounds contained in the essential oil and mastic water are obtained by the distillation of mastic gum. The essential oil, derived from CGM, approximately 3% of mastic gum dry weight, constitutes the most studied part of *Pistacia*, and its chemical composition varies and depends on the gum quality that is influenced by its purity, the collection period, and the time elapsed between exudation from the trunk and collection. Essential oil is approximately composed of monoterpene hydrocarbons (50%), oxygenated monoterpenes (20%), and sesquiterpenes (25%) [50,51,53]. Several research groups have widely studied the chemical composition of essential oil, mainly by GC-MS [53,54,55,56,57]. Approximately 69–72 constituents have been identified in essential oil (Table 1), where α-pinene (30–75%), myrcene (3–60%), and β-pinene (1–3%), are the major components, and, together, they constitute about the 90% of the oil [50,51,53,55,56]. Different conditions of receipt or storage of the essential oil may influence the chemical composition.

CGM essential oil is typically produced by steam and/or water distillation. Supercritical fluid extraction (SFE), with the utilization of environmentally friendly solvents (supercritical CO_2_ and ethanol), has been recently developed as an alternative green method [57]. The volatile part of the resin obtained by this technique presents some differences in the composition compared to essential oil produced by hydro-distillation [57]. Probably the most important advantage of SFE is the low temperature of the process avoiding thermal degradation of the most sensitive compounds.

**Figure 6:**
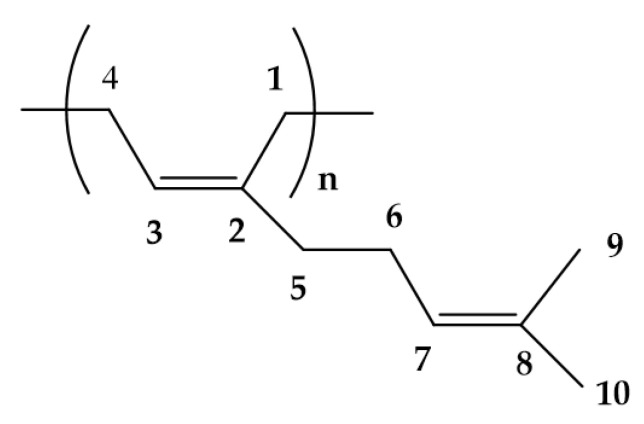
The monomeric base unit of CMG’s polymer: *cis*-1,4-poly-β-myrcene.

Finally, other compounds belonging to miscellaneous chemical classes (~5%) are also present in the insoluble and sticky polymer, forming the resin structure (Figure 6), which constitutes about 25–30% of the dry weight. The main component of the polymer consists of poly-β-myrcene, predominantly present in the cis conformation [58], with the molecular weight distribution broad and matched up to 100,000 Da.

Contrary to fruit and leaves rich in polyphenols, CMG contains traces of phenolic compounds, mainly phenolic acids. Methanol/water extraction, HPLC fractionation, and GC-MS analyses demonstrated the presence of tyrosol, p-hydroxy-benzoic, p-hydroxy-phenylacetic, vanillic, gallic, and trans-cinnamic acids [59]. The presence of α-tocopherol in CMG has also been reported [60].

## 4. Inflammatory Bowel Disease

**Figure 7:**
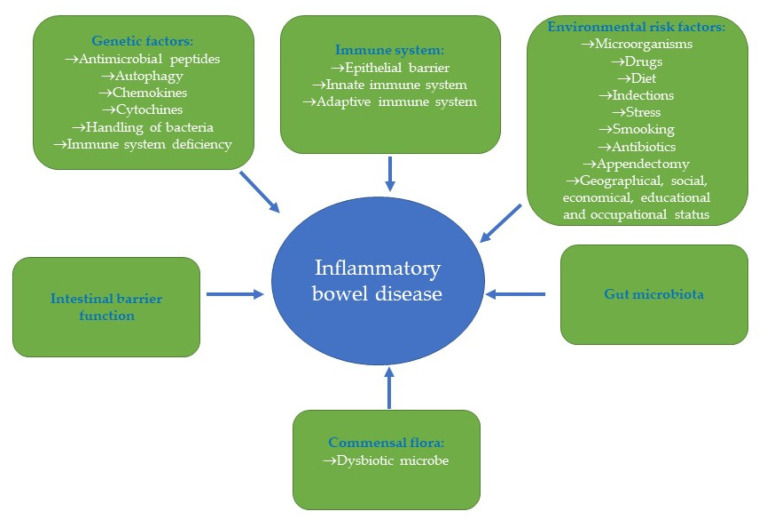
Multifactorial pathogenesis of IBD.

IBD is a group of intestinal disorders, mainly represented by Crohn’s disease (CD) and ulcerative colitis (UC), which are characterized by chronic inflammation of the gastrointestinal tract. Both CD and UC share the same clinical course. Differences are confined to the site and nature of the inflamed lesions [61,62]. IBD affects millions of people worldwide and significantly increases the incidence of colorectal cancer [63]. However, the precise etiology and pathogenesis of IBD are not fully understood. The pathogenesis of IBD is multifactorial, including genetic predisposition, immune dysregulation, barrier dysfunction, and altered microbial flora, as well as environmental and lifestyle factors (Figure 7).

**Figure 8:**
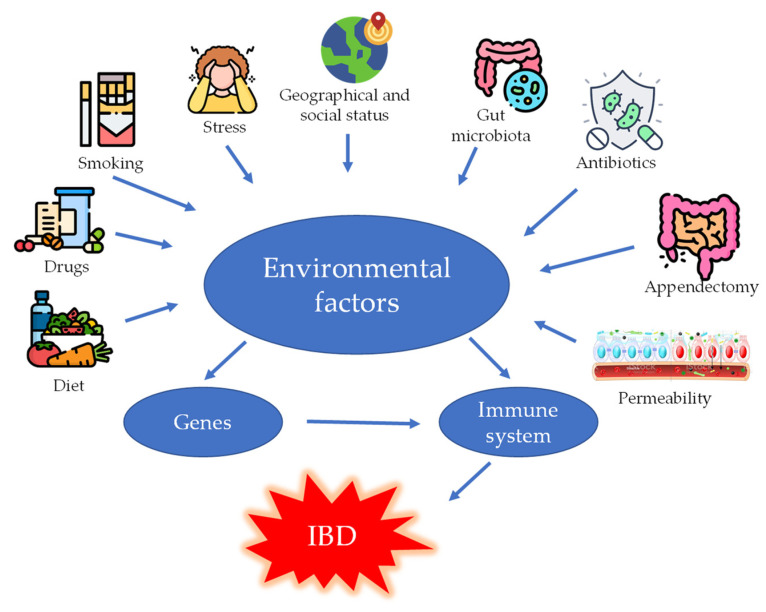
Environmental factors affecting IBD.

Genetic predisposition contributes to the dysregulation of both innate and adaptive immunity [64]. Environmental triggers such as diet, infection, antibiotics, smoking, drugs, and toxin exposure affect the intestinal microbiome and influence epigenetic changes that alter immune regulation [65]. Then, IBD results from an extremely complex interaction between all of the aforementioned factors (Figure 8) that make prevention and treatment of IBD challenging. While treatment for IBD patients has improved over the past two decades, not all patients are responding to available therapies. For this reason, there is still a great need to further expand the therapeutic repertoire in an effective way for patients with IBD [66].

Treatment, particularly in patients with more aggressive disease, is generally lifelong and often involves the use of corticosteroids, immunosuppressants, and antitumor necrosis factor (TNF) antibodies and surgery. Faced with such complexity in the drug treatment of IBD, combined with the economic burden and inadequate response to available drugs or concerns about side effects, researchers have begun to focus on the globally accepted complementary and alternative medicines for their unique effectiveness and moderate cost.

According to the World Health Organization (WHO) report, more than 80% of the world’s population relies on the traditional system of medicine for their health. Traditional medicines, mainly herbal products, have shown efficacy in experimental models and clinical trials of IBD in (i) maintaining intestinal epithelial barrier integrity, (ii) regulating macrophage activation, (iii) modulating innate and adaptive immune responses, and (iv) inhibiting TNF-alpha activity. Herbal medicinal plants have long been used as an alternative therapy to prevent and treat various diseases, including a wide range of acute and chronic gastrointestinal disorders [67,68,69,70,71,72]. In particular, the use of the *Pistacia* species has been covered extensively [5,47,72,73,74,75]. Considering the three principal families of CMG chemical components, free fatty acids, terpenes, and phenolic compounds, literature data could support the beneficial effects of *P. lentiscus* on IBD. The inverse correlation of IBD severity to ω-3-PUFAs intake [76] and the anti-aging effect of ω-3-PUFAs supplementation on the microbiome are known [77]. In contrast, the excessive consumption of the ω-3 linoleic acid may promote the development of IBD, increasing proinflammatory eicosanoids and dysregulating the intestinal endocannabinoid system [78]. Terpenes have never been clinically studied in IBD patients, but various molecules of this family have been evaluated in different IBD models promoting them as promising candidates for IBD treatment. Terpenes investigation in IBD is based on their safety, pharmacokinetics, and thanks to their ability to regulate inflammatory pathways involving interleukins IL-1β, IL-6, and IL-8, eicosanoids, and growth factors [79]. Starting from their ability to modulate cellular signaling pathways and transcription factors important in IBD progression such as proinflammatory interleukins and eicosanoids or TNF-alpha production, polyphenols also have been assessed as candidate treatments in IBD animal models. Together with their anti-inflammatory and antioxidant effects, polyphenols are also able to modulate the gut microbiome, promoting commensals and inhibiting pathogens [79].

In the present review, we will only deal with the use of *P. lentiscus* for the treatment of IBD.

### Effects of Pistacia lentiscus on IBD

All of the studies regarding *P. lentiscus* experimentation in IBD animal models or clinical trials are summarized in Table 2.

***Animal Models.*** The anti-inflammatory effects of *P. lentiscus* observed in clinical studies were demonstrated by applying the trinitrobenzene sulfonic acid (TNBS)-induced colitic rat model [80]. This model is particularly useful for studying biochemical inflammatory pathways because is quite similar to CD in humans with elevated levels of T-helper 1 cytokines such as TNF-α. TNF-α is considered the key molecule, shown by the fact that when mice with TNBS-induced colitis were treated by intraperitoneal injection of antibodies to TNF-α, improvements of both the clinical and histopathological signs of disease were found [81]. This study suggests that *P. lentiscus* in the form of powder at 100 mg/kg of body weight reduces, via downregulation, the production of inflammatory cytokines TNF-α, ICAM-1, IL-6, and IL-8 [80]. Also, colon damage was significantly reduced. In addition, to evaluate oxidative stress, malonaldehyde concentration in colonic tissues was measured and was significantly suppressed in all treated groups. These results demonstrate that the powder of *P. lentiscus* exerts a beneficial effect in severe TNBS-induced colitis in rats. A possible mechanism proposed by the authors could be the scavenging of free radicals and the regulation of key inflammatory mediators of IBD by the terpenes and phenolic compounds present in *P. lentiscus* resin [80]. The mastic powder mixture (100 mg/kg of body weight) or the respective powder mixture components (CM) and acidic (AF) and neutral fractions (NF) of the powder [48] or of oleanolic acid (OA, a major triterpenic acid found in CM) [82] were individually applied to the above experimental model of colitis (TNBS), and levels of TNF-α, IL-6, IL-8, and ICAM-1 were measured. Histological improvement of colitis and a significant regulation in inflammation occurred with the CM powder mixture, while no histological improvement was observed with AF and NF, although it reduced the levels of inflammatory markers [83].

In an attempt to elucidate the mechanism of the anti-inflammatory activity in experimental colitis, the same authors investigated the mechanism underlying this effect in a model of inflammation in co-cultured human colon epithelial HT29 cells and monocytes/macrophages. Results from the in vitro experiments pointed towards a downregulation of IL-8 and nuclear factor jB p65 (NF-jB p65) with mastic powder and a reduction in lactate dehydrogenase release. Neither fractions nor OA were the sole bioactive component. Most probably, the mastic powder rather than its individual fractions exerts an anti-inflammatory activity via NF-B regulation [83].

In experimental colitis induced by 2,4,6-trinitrobenzenesulfonic acid, the curative and preventive effects of mastic oil, 38.8% by weight of *P. lentiscus* fruit, were examined. When mastic oil was added, macerated, and mixed with a standard diet at a dose of 30 mg of oil/100 g of feed/rat, a protective effect on intestinal inflammation was observed against weight loss, rectal bleeding, and diarrhea. This beneficial effect involves a modification of the metabolism of arachidonic acid [84]. When rats with experimental colitis, induced by administering 3% acetic acid intra-rectally, were treated with *P. lentiscus* mastic oil, administered intra-rectally, a statistically significant decrease in TNF-α level after 7 days was observed, while IL6 did not change [85].

More recently, the anti-inflammatory effect in vivo of *P. lentiscus* was shown in a rat model of UC inflammation. The anti-inflammatory action of the aqueous extract of *P. lentiscus* leaves used in experimental colitis induced by dextran sulfate sodium-induced acute colitis is attributed to (i) the cellular level by inhibiting immune cell activation and recruitment, (ii) the membrane level by blocking proinflammatory cytokine receptors, or (iii) the intracellular level by reducing NF-κB expression and (inducible Nitric Oxide Synthase) iNOS and the production of proinflammatory cytokines [86].

A study investigating the therapeutic action of a combination of *P. atlantica* subspecies *Kurdica* oleo-gum-resin and honey in acetic-acid-induced colitis in rats demonstrated that this combination induced significant improvements in macroscopic and microscopic scores. Colonic levels of myeloperoxidase, IL-6, and TNF-α decreased significantly in rats treated with the mixture, while a significant decrease in (Toll-like receptor) TLR-4 mucosal gene expression and a significant improvement in colitis were observed. Furthermore, its reduction in gut inflammation and colon ulcer severity was evidenced by the downregulation of inflammatory cytokines, decreased neutrophil infiltration, and suppressed TLR-4 expression [87].

Finally, the effects of masticadienonic acid (MDA), one of the most abundant constituents isolated from CMG, were evaluated using a dextran sulfate sodium-induced acute colitis mouse model [88]. MDA ameliorates the severity of IBD by increasing the body weight and colon length, the disease activity index, and the histological score. MDA treatments reduce the release of serum inflammatory cytokines TNF-α, IL-1β, and IL-6. MDA supplementation could also improve the intestinal barrier function by activating the NF-E2-related factor-2 (Nrf2) signaling pathway and restoring the expression of tight junction proteins zonula occludens-1 (ZO-1). The Nrf2 signaling pathway is involved in the transcriptional regulation of tight junction proteins and improves barrier function in colitis mice. In addition, MDA administration modulates the gut microbiota composition. In IBD, in the presence of an antigen, activated macrophages lead to the production of TNF-α. Furthermore, the expression of several cytokines, including IL-6, IL-8, and IL-1β, as well as intercellular adhesion molecule-1 (ICAM-1), is mediated by the activation of nuclear factor-κB (NF-κB). Consequently, suppressing the production of these pro-inflammatory mediators has therapeutic potential in IBD treatment. CMG treatment in variously induced experimental colitis described above suggests that it has an anti-inflammatory capacity by limiting the production of pro-inflammatory factors, driven by the downregulation of NF-κB signaling pathways. Further, it regulates the oxidant/antioxidant balance and alleviates intestinal damage and inflammation of ulcerative colitis. The induction of a protective barrier effect, by reducing paracellular permeability in human colon cell models, has also been demonstrated. In the most recent paper [88], the same mechanism has been demonstrated in colitis mice via the activation of the Nrf2 signaling pathway and upregulation of tight junction proteins.

***Clinical trials.*** Data about resin’s effects on IBD are still limited. Based on the anti-inflammatory properties observed in animal models and in vitro studies, *P. lentiscus* has been suggested for the treatment of IBD. A number of preliminary clinical trials have been carried out. The first study evaluated the efficacy of CMG on the clinical course and plasma inflammatory mediators of patients with active Crohn’s disease (CD). In a small clinical study including 10 patients with mild or moderately active CD, recruited for a 4-week treatment with mastic caps (2.2 g/day), it was demonstrated that CMG was effective in the regulation of inflammation, evaluated by C-reactive protein (CRP), IL-6, TNF- α, and MCP-1 in plasma, as well as in the regulation of oxidative stress, evaluated by total antioxidant potential. CMG treatment significantly decreased the CD Activity Index (CDAI), which probably occurred through the decrease in the pro-inflammatory IL-6 and the increase in the total antioxidant potential, inducing remission in seven out of ten patients [89]. In addition, the nutritional risk index (NRI), one of the most useful measures of nutritional status incorporating albumin level and body weight, has been improved. In particular, the main NRI element that showed improvement was body weight gain, attributed to the decrease in loose stools with the consequent improvement in nutrient absorption, as the daily energy intake remained unchanged during the study [89]. No significant side effects were reported. In the same cohort of patients, the authors demonstrated that mastic administration influences the secretion of cytokines by peripheral blood mononuclear cells (PBMCs). In this study, mastic showed immunomodulator activity on PBMCs, acting as an inhibitor of TNF-α and a stimulator of macrophage migration inhibitory factor [90], suggesting an additional inhibitory mechanism of monocyte chemotaxis and thus providing more support to the role of CMG as an immune system regulator.

In 2019, Papada et al. [91], based on the findings of the pilot study by Kaliora et al. [87,88], performed a randomized controlled trial to further investigate the effects of CMG on patients with IBD. The authors’ primary aim was a clinically meaningful improvement in patients’ quality of life assessed by means of the Inflammatory Bowel Disease Questionnaire (IBDQ), which consists of 32 questions on the bowel, social, systemic, and emotional performances [92]. Secondary outcomes included improvement in fecal and serum inflammatory markers as assessed with the measurement of fecal lysozyme, calprotectin, lactoferrin, serum IL-6 and IL-10, and CRP at baseline and follow-up. In addition, improvement in biochemical indices associated with the nutritional state, serum Fe, and albumin or acute phase reactants, such as plasma fibrinogen, were determined. A total of 60 patients with endoscopy-proven UC or CD were randomly administered with CMG 2.8 g/day or placebo for 3 months in addition to stable medical treatment. Patients treated with mastic had a significant decrease in fecal lysozyme compared to patients on placebo. This finding, in light of previous research that suggested that fecal lysozyme is increased in patients with IBD [93], is indicative of lower disease activity. In addition to this, a significant improvement in IBDQ scores, reflecting a beneficial effect on patients’ quality of life, was observed in the mastic arm compared to the baseline.

In a randomized, double-blind, placebo-controlled study, the antioxidant efficacy of a *P. lentiscus* supplement in IBD was evaluated [94]. Furthermore, the profile of free amino acids in plasma (AA) has been characterized in patients with CD (40) and UC (20). A total of 60 patients were randomly assigned to either *P. lentiscus* supplement (2.8 g/day) or placebo for three months, and oxidized low-density lipoprotein (oxLDL), oxLDL/HDL (high-density lipoprotein), and oxLDL/LDL decreased significantly in the intervention group, confirming its antioxidant activity. Several changes were reported in AA levels. The general tendency for plasma AA to increase in placebo-treated UC patients may indicate increased de novo synthesis of AA in the presence of inflammation. The fact that AA changes were predominantly present in the UC patient group may indicate a more favorable effect of *P. lentiscus* in active UC patients.

Based on the need for effective maintenance treatment without serious side effects and on the key role of AA in remission maintenance, Papada et al. [95,96] aimed to investigate the effects of CMG on the clinical course and AA profile of IBD patients in remission. AA, participating in gene expression, intracellular protein turnover, protein synthesis, oxidative stress, and the stimulation of lymphocyte proliferation, inflammatory cytokines production, and T cell-mediated immunity [94], plays a key role in pathways regulating intestinal health. AAs are used as therapeutic options in order to maintain intestinal integrity in IBD [97]. A total of 68 IBD patients in clinical remission in the last 6 months were randomly treated with mastic (2.8 g/day) or placebo adjunct to standard medication with the effect of CMG inhibition of plasma-free AA increase. In addition, patients treated with placebo, in contrast to patients with CMG, presented an increase in serum IL-6 or in fecal biomarkers of IBD activity such as calprotectin and lactoferrin. These data could demonstrate the need for de novo synthesis of AA in patients with increased inflammation depicted by increased IL-6, fecal calprotectin, and lactoferrin. Since changes in AA levels are considered an early prognostic marker of the disease activity, this indicates a potential role of CMG in remission maintenance. More recently, Amerikanou et al. [98] investigated a CMG regulatory effect on IL-a17A serum levels in IBD patients and, as a functional readout of microbial activity, the alterations of the fecal metabolome. In this study, patients with a diagnosis of IBD, 43 UC and 86 CD, either in remission (*n* = 67) or in relapse (*n* = 62), were recruited. The CMG group received natural mastic at a dose of 2.8 g daily, while the placebo group received identical placebo tablets for 6 months for patients in remission and for 3 months for patients in relapse. Levels of interleukin-17A increased significantly in the CMG group, and the mean change differed significantly between groups. Fecal metabolomics indicated that CMG can affect the metabolic profile of IBD patients in remission, increasing the serum levels of glycine and tryptophan. Glycine has been proposed to have a therapeutic effect against IBD, while tryptophan derivatives are involved in immunoregulatory mechanisms, such as the Th17 cell differentiation. These data support the hypothesis that the immunoregulatory role of CMG in quiescent IBD involves the regulation of Th17 cells’ function and differentiation.

In conclusion, the data from the literature show that CMG reduces pro-inflammatory cytokines such as IL-6 [89] and TNF-α [90] and increases the levels of interleukin-a17A [98], which is considered a protective key factor in the development and relapse of IBD. These data have been corroborated by randomized controlled studies showing that *P. lentiscus* may also reduce free AA in plasma [94], a surrogate for inflammation and cell homeostasis [96], and may play a key role in pathways regulating intestinal health.

On account of these data, it has been argued that CMG may be used as a supplement to decrease disease activity, improve nutritional status, and maintain clinical remission in IBD patients.

Unfortunately, despite the large amount of preliminary data on the effect of *P. lentiscus* on biochemical markers of inflammation and homeostasis, the scientific evidence of its clinical effectiveness in IBD is still scanty and mainly based on a few randomized controlled studies. These studies showed that *P. lentiscus* may improve IBD quality of life, although to the same extent as placebo, and its effects on IBD activity, assessed by scores tools, although with some benefits, still remain uncertain [91]. However, it should be acknowledged that the sample sizes of these trials are small and that the true extent of *P. lentiscus’s* potential benefit is difficult to assess because it has been associated with different drugs, as usually happens for most supplementary treatments. Therefore, large prospective trials are still needed.

**Table 2:** *Pistacia lentiscus* mastic gum experimentation in inflammatory bowel disease.

Study (Authors, Year)	Design	Effect
***Animal Models***		
**[80] Giovxari et al., 2011**	-Colitic rat model induced with TNBS assigned to seven groups: A, control; B, colitic; C–F, colitic rats treated daily with PL powder at 50, 100, 200, and 300 mg/kg/day, respectively; and G, colitic rats treated daily with cortisone (25 μg/kg)	-TNF-α, ICAM-1, IL-6, IL-8, and IL-10 ↓ -Damage ↓ -Malonaldehyde ↓
**[83] Papalois et al., 2012**	-Colitic rat model induced with TNBS -Treated with Chios mastic (CM) powder at 100 mg/kg/day or the respective CM components: inulin (40 mg/kg/day); acidic fraction (AF- 24 or 48 mg/kg/day); neutral fractions (NF- 24 or 48 mg/kg/day); and oleanolic acid (OA-14 mg/kg/day) for 5 days	-TNF-α, ICAM-1, IL-6, and IL-8, ↓ -CM: histologic improvement and regulation of inflammation -AF and NF: no histologic improvement, inflammatory markers reduction
**[84] Naouar et al., 2016**	-Colitic rat model induced with TNBS -Treated with PL mastic oil at a 30 mg of oil/100 g of feed/rat/day for 2 months before colitis induction	-Weight loss, rectal bleeding, diarrhea, ulceration, hyperplasia, and cryptitis ↓
**[85] Ostovan et al., 2020**	-Control, colitis without treatment, and colitis induced with 3% acetic acid rat models were treated with (i) PL mastic oil, 400 mg/kg/daily, administered orally or intra-rectally; (ii) prednisolone 5 mg/kg/day; or (iii) sesame oil 2 mL/kg/day for 7 days	-TNF-α ↓ (as prednisolone) -= IL6 (as sesame oil)
**[86] Boutemine et al., 2021**	-Control, colitis without treatment, and colitic induced with 3% dextran sulfate sodium rat models were treated with aqueous extract of PL leaves 50, 100 or 200 mg/Kg/day, respectively, for 7 days	-Activation and recruitment of immune cells ↓ (cellular level) -Blockade of pro-inflammatory cytokine receptors (membrane level) -NO, IL-6, and TNF-α ↓ -Pro-inflammatory cytokines (intracellular level)
**[88] Cui et al., 2023**	-Acute colitis mouse model, induced with dextran sulphate sodium -Colitic rat group were treated with Masticadienonic acid (MDA), one of the most abundant constituents isolated from Chios mastic gum, solubilized with 30% PEG-400 at (i) low-dose MDA, 10 mg/kg/day, or (ii) high-dose MDA, 100 mg/kg/day, for 14 days.	-Body weight, colon length, disease activity index, and histologic score ↓ -TNFα, IL-1β and IL-6 ↓ -Intestinal barrier function by Nrf2 ↑ _ restoring ZO-1 and occluding tight junction proteins -Modulation of the composition of the intestinal microbiota
***Cellular model***		
**[81] Papalois et al., 2012**	-Inflammation model in co-cultured human colon epithelial HT29 cells and monocytes/macrophages	-IL-8 and NF-jB p65 ↓ -LDH↓
***Clinical Trials***		
**[89] Kaliora et al., 2017**	-10 patients with active CD and 8 healthy controls -Treated with mastic caps 6 caps/day, 0.37 g/cap for 4 weeks	-CD activity index ↓ -IL-6 and CRP ↓ -TNF-α and MCP-1 ↓ not significant -Total antioxidant potential ↑
**[90] Kaliora et al., 2017**	-10 patients with active CD and 8 healthy controls -Treated with mastic caps, 6 caps/d, 0.37 g/cap for 4 weeks	-TNF-α secretion in PBMC ↓ -MIF ↓ PBMC -No significant changes in IL-6, MCP-1
**[94] Papada et al., 2018** **[94] Papada et al., 2019**	-60 patients with IBD -Treated with 2.8 g of mastic daily for 3 months or placebo randomized	-Improvement in IBDQ - oxLDL ↓ -Plasma cysteine and fecal lysozyme ↓ -No impact on serum IL-6, fecal calprotectin and fecal lactoferrin
**[98] Amerikanou et al., 2021**	-129 patients with IBD—67 randomized to mastic group and 62 to placebo -Treated with 2.8 g daily for 6 months for patients in remission and for 3 months for patients in relapse	IL-17A ↑

Abbreviations: tumor necrosis factor-α (TNF-α), intercellular adhesion molecule-1 (ICAM-1), interleukins (IL) IL-6, IL-8, trinitrobenzene sulfonic acid (TNBS), NF-E2-related factor-2 (Nrf2), zonula occludens-1 (ZO-1), lactate dehydrogenase (LDH), C-reactive protein (CRP), peripheral blood mononuclear cells (PBMCs), macrophage migration inhibitory factor (MIF), Inflammatory Bowel Disease Questionnaire (IBDQ).

## 5. Conclusions

Despite the great progresses in the field of human health and the remarkable development of medical products, natural supplements still stimulate medical research interests. In this context, existing literature suggests that Chios mastic possesses anti-inflammatory and antioxidant properties promoting it for the treatment of various diseases and, in particular, IBD. The limited number of research data in IBD, however, comes from studies on experimental animal or cellular models, and the number of human studies in this direction is, for the time being, scant. Although the findings were encouraging, more research is necessary to further validate the effectiveness of mastic, and further clinical research is needed to evaluate the therapeutic potential of mastic in IBD. Larger randomized controlled studies with a homogeneous group of patients (i.e., relapse or remission) and providing a longer treatment period could achieve this aim. Moreover, the mechanism of action of CMG is complex to understand probably due to the complexity of CMG’s chemical composition. Mechanistic aspects are mostly investigated in cellular models, while clinical trials only analyzed some inflammatory or oxidative stress biomarkers together with IBD symptoms. This literature revision highlights the lack of a phytochemical investigation associated with a clinical study that could help to pave the way for CMG mechanism of action elucidation.

## Figures and Tables

**Table 1:** Phytoconstituents of CMG.

Essential Oil

*Monoterpene hydrocarbons*
α-Pinene, β-pinene, β-myrcene, tricyclene, camphene, verbenene, 2-methylanisole, *p*-cymene, limonene, *trans*-linalool oxide, α-campholene aldehyde, *trans*-pinocarveol, *trans*-verbenol, pinocamphone, pinocarvone, *p*-mentha-1,5-dien-8-ol, myrtenal, myrtenol, verbenone, β-caryophyllene, α-caryophyllene, caryophyllene oxide, 28-nor-12,17-oleanadien-3-ol, lupenone, tirucallone, tirucallol, dammaradienol, 3-methoxy-28-norolean-12-ene, β-amyrone, 28-norolean-17-en-3-ol, 28-norolean-17-en-3-one, 6-methyl-28-norolean-17-en-3-one, olean-18-en-3-one, β-amyrin, 28-nor-12,17-oleanadien-3-one, oleanenone derivative, dammarane derivative, hydroxydammarenone, oleanonic aldehyde, moronic aldehyde, 28-nor-12,18-oleanadien-3-ol, and isomasticadienolic aldehyde
*Oxygenated monoterpenes e Benzenoids*
Perillene, α-linalool, camphenol, α-campholenal, pinocarveol, *cis*-verbenol, verbenol, verbenone, bornyl acetate, campholene, camphor, 3,6,6-trimethyl norpinan-2-one, pinocarvone, *cis*-3-pinanone, *cis*-carveol, 1-ethenyl-2,4-dimethylbenzene (or 1-Methyl-4-(2-propenyl)-benzene), *o*-methyl-anisole, *o*-cymene, *m*-cymene, *p*-cymene, β-methyl-cinnamaldehyde, myrtenal, *p*-cymen-8-ol, carvone, and trimethyl-hydroquinone
*Sesquiterpene hydrocarbons*
β-Caryophyllene, α-humulene, α-longipinene, α-ylangene, α-copaene, β-bourbonene, β-elemene, isocaryophyllene, α-muurolene, and D-germacrene
*Oxygenated sesquiterpenes*
Caryophyllene oxide, α-humulene epoxide, and 3,8,8-trimethyl-1,2,3,4,5,6,7,8-octahydro-2-naphthalenyl methyl acetate
**Triterpenes**
*Pentacyclic triterpenes*
Oleanonic acid, oleanolic acid, moronic acid, oleanonic aldehyde, oleanolic aldehyde, 28-nor-oleanone, 28-nor-oleanole, β-amyrine, β-amyrone, 28-hydroxy-β-amyrone, germanicol, lupeol, betulonal, lup-20(29)-ene-3-one, 3-oxo-28-norlup-20(29)-ene
*Tetracyclic triterpenes*
24*Z*-Masticadienonic acid, 24*Z*-isomasticadienonic acid, 24*Z*-masticadienolic acid, 24*Z*-isomasticadienolic acid, mastichadienolal, isomastichadienolal, tirucallol, dammaradienone, mastichinoic acid, butyrospermol, dipterocarpol, and 20S-3β-acetoxy-20-hydroxydammar-24-ene
*Trycyclic triterpenes and bycyclic triterpenes*
3β-Hydroxymalabarica-14(26),17*E*,21-triene, 3-oxomalabarica-14(26),17*E*,21-triene, (8*R*)-3β,8-dihydroxy-polypoda-13*E*,17*E*,21-triene, and (8*R*)-3-oxo-8-hydroxy-polypoda-13*E*,17*E*,21-triene.
**Polyphenols**
Tyrosol, p-hydroxy-benzoic, p-hydroxy-phenylacetic, vanillic acid, gallic acid, and *E*-cinnamic acid.
**Others**
3-Ethylidene-1-methylcyclopentene, methyl-o-cresol, 1-dodecanol, 2,5-dimethoxytoluene, 3,5-dimethoxytoluene, (*E*)-anethole, 2-undecanone, octyl formate, 2-methyl-3-buten-2-ol, pinanediol, *trans*-linalool oxide, *cis*-linalool oxide, 6,7-dihydro-7-hydroxylinalool, 5,5-dimethyl-2(5H)-furanone, α-irone, o-methylanisol, methyleugenol, methylisoeugenol, α-fenchyl acetate, 4-acetyl-1-methylcyclohexene, and 2-undecanone

## Data Availability

Not applicable.
